# Alterations of the Immunologic Co-Stimulator B7 and TNFR Families Correlate with Hepatocellular Carcinoma Prognosis and Metastasis by Inactivating STAT3

**DOI:** 10.3390/ijms20010156

**Published:** 2019-01-03

**Authors:** Yi-Ming Li, Zhen-Yu Liu, Zhu-Chun Li, Jian-Chao Wang, Jing-Min Yu, Hai-Jiao Yang, Zhi-Nan Chen, Juan Tang

**Affiliations:** 1 National Translational Science Center for Molecular Medicine, Xi’an 710032, China; sydlym@163.com (Y.-M.L.); qqqzhenyu20052008@163.com (Z.-Y.L.); lizhuchun2011@hotmail.com (Z.-C.L.); wangjianchao-999@163.com (J.-C.W.); yujingmin1226@163.com (J.-M.Y.); yhjjqq.love@163.com (H.-J.Y.); 2 Cell Engineering Research Center & Department of Cell Biology, School of Basic Medicine, Fourth Military Medical University, Xi’an 710032, China

**Keywords:** immune landscape, liver cancer, metastasis

## Abstract

Blockade of the immunosuppressive checkpoint receptors cytotoxic T-lymphocyte-associated protein 4 (CTLA4) or programmed death 1 (PD-1) and its cognate ligand, programmed death 1 ligand (PD-L1), has altered the landscape of anti-tumor immunotherapy. B7 family and tumor necrosis factor receptor (TNFR) superfamily play a crucial role in T cell activation, tolerance, and anergy through co-stimulatory and inhibitory signal transduction. Investigating the immune molecular landscapes of the B7 and TNFR families is critical in defining the promising responsive candidates. Herein, we performed comprehensive alteration analysis of the B7 and TNFR family genes across six hepatocellular carcinoma (HCC) datasets with over 1000 patients using cBioPortal TCGA data. About 16% of patients had both B7 and TNFR gene alterations. TNFR gene amplifications were relatively more common (1.73–8.82%) than B7 gene amplifications (1.61–2.94%). Analysis of 371 sequenced samples revealed that all genes were upregulated: B7 and TNFR mRNA were upregulated in 23% of cases (86/371) and 28% of cases (105/371), respectively. Promoter methylation analysis indicated an epigenetic basis for B7 and TNFR gene regulation. The mRNA levels of B7 and TNFR genes were inversely correlated with promoter methylation status. B7-H6 expression was significantly associated with worse overall survival, and B7-H6 mRNA was increased gradually in cases with gene copy number alterations. B7-H6 overexpression was associated with aggressive clinicopathologic features and poor prognosis in HCC. Downregulation of B7-H6 in HCC cells significantly inhibited cell adhesion, proliferation, migration, and invasion. Knockdown of B7-H6 in HCC cells inhibited tumor growth and metastasis in vivo. B7-H6 promoted HCC metastasis via induction of MMP-9 expression and STAT3 activation. B7-H6 and STAT3 performed functional overlapping roles on enhancing the MMP-9 promoter activity in HCC cells. These results suggest that alterations of the immunologic co-stimulator B7 and TNFR families correlate with HCC metastasis and prognosis, and especially B7-H6 plays a critical role in promoting metastasis of HCC.

## 1. Introduction

Hepatocellular carcinoma (HCC) is one of the leading causes of cancer-related death worldwide [1]. The development and progression of HCC is driven by both intrinsic epigenetic alterations and extrinsic tumor/T-cell crosstalk [2,3]. Accumulating evidence has demonstrated that tumor immune microenvironment facilitates tumor cells to immune escape by the infiltrating suppressive immune cells [1,4]. In recent years, inhibitory checkpoints—including cytotoxic T lymphocyte-associated antigen 4 (CTLA-4), programmed cell death protein-1 (PD-1), and programmed cell death ligand 1 (PD-L1)—have been recognized to suppress anti-tumor immune responses in solid tumors [5]. Blockade of these immune checkpoints has been translated into effective strategies for cancer immunotherapy. Despite that therapeutic blockade has revealed durable antitumor responses and long-term remissions, immune escape, or resistance to immunotherapy contributes to the relatively low response rates in HCC patients [2,6]. Thus, the best candidates that benefit from immune checkpoint therapy are much warranted but still unclear.

It is well known that T cells play a critical role in regulating immune responses and are activated by two classical signals: antigen recognition (signal 1) characterized by T cell receptors (TCRs) engaging major histocompatibility complex (MHC)-embedded antigen peptides (pMHCs) within the contact region; and co-stimulation (signal 2) which requires a combination of several co-regulators consist of both co-stimulatory and co-inhibitory molecules [7]. Therapeutic modulation of these signal transduction pathways has been translated into effective strategies for cancer immunotherapy [8]. At present, the co-stimulatory and co-inhibitory pathways mainly contain two major families: the B7 family of immunoglobulins3 and the tumor necrosis factor receptor (TNFR) superfamily [9,10].

The B7 family has 10 reported members including CD80 (B7-1), CD86 (B7-2), B7-DC, PD-L1 (B7-H1), B7-H2, B7-H3, B7-H4, B7-H5, B7-H6, and B7-H7 [9]. Moreover, CD80 (B7-1), B7-2 (CD86)/CTLA-4, and B7-H1 (PD-L1)/PD-1 have been identified as promising targets and their inhibitors have achieved great success in cancer immunotherapy [11]. The TNFR superfamily proteins are expressed by antigen-presenting cells (APC) or tumor cells, including TNFSF4 (OX40L), TNFRSF5 (CD40), TNFSF7 (CD70), TNFSF9 (CD137L), TNFRSF14 (HVEM), and TNFSF18 (GITRL) [10]. TNFR superfamily proteins function as secondary T cell activation signals and may serve as novel candidates in cancer immunotherapy. However, the systemic alterations of these families have not been defined in HCC.

The B7 and TNFR family members and their receptors exhibit great potential as therapeutic targets in HCC immunotherapy. In this study, we elucidate comprehensive molecular profiling of the 10 B7 and 6 TNFR family genes across 6 HCC studies by TCGA data from cBioPortal for Cancer Genomics. Herein, we analyzed the mutation, amplification, copy number data, mRNA dysregulation, DNA methylation and the relevant clinical profiles to understand the molecular landscape and immunotherapeutic implications of the B7 and TNFR families in HCC. By bioinformatics, we focused on B7-H6 and further investigated the effect of B7-H6 on the metastasis and prognosis of HCC, aiming to provide novel efficacious therapeutic targets that stratify patients responsive to HCC immunotherapy.

## 2. Results

### 2.1. Gene Alterations of B7 and TNFR Family Across Liver Cancer Studies

**Figure 1:**
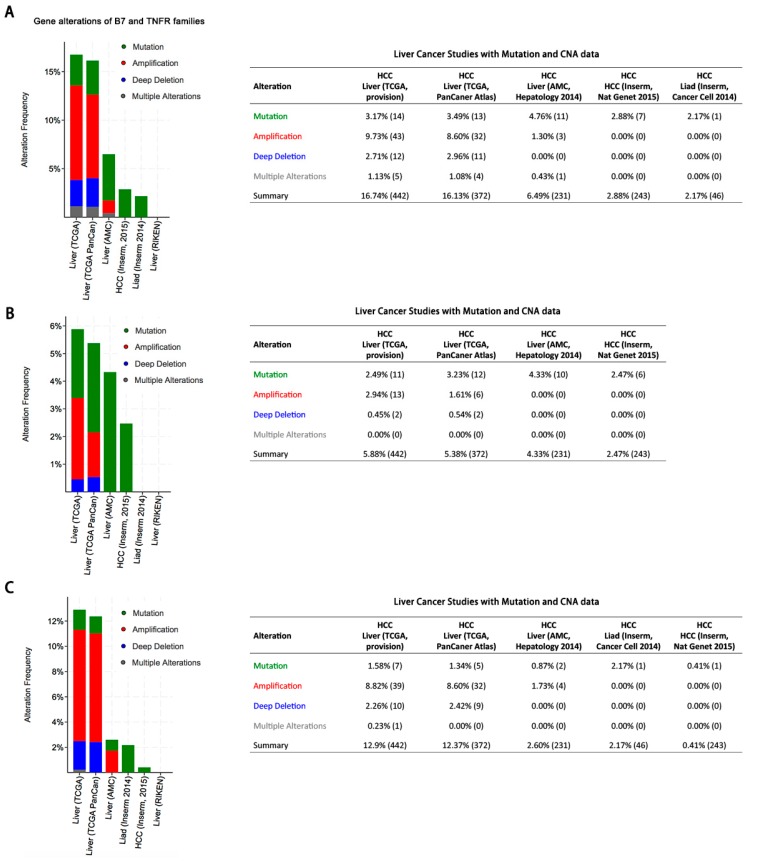
Gene alteration frequencies in the B7 and TNFR families across six HCC studies. (**A**) Gene alterations of B7 and TNFR families. (**B**) Gene alterations of B7 family. (**C**) Gene alterations of TNFR family.

It has been well recognized that B7 and TNFR family molecules play crucial roles in crosstalk between immune cells and tumor cells [12,13]. However, the wide gene alterations of B7 and TNFR family molecules remain unclear in HCC. Herein, we enrolled six large-scale HCC studies and investigated the frequencies of B7 and TNFR gene alterations including mutations, amplifications, and deletions. In two studies (TCGA and TCGA PanCan), both B7 and TNFR gene alterations were observed in about 16% of patients (Figure 1A). For B7 family genes, frequencies of mutations (2.47–4.33%) were similar to copy number alterations (CNA), including amplifications and deep deletions (a total of 2.15–3.39%; Figure 1B). To be specific, the amplification rates (0.00–2.94%) were higher than deletion rates (0.00–0.45%) in B7 gene family molecules (Figure 1B). For TNFR family genes, the frequencies of mutations (0.41–2.17%) were lower than copy number alterations (CNA), including amplifications and deep deletions (a total of 1.73–11.08%; Figure 1C). To be specific, the amplification rates (0.00–8.82%) were higher than deletion rates (0.00–2.42%) in TNFR gene family molecules (Figure 1B). In addition, amplifications were relatively more common (0.00–8.82%) in TNFR gene family than those of B7 gene family (0.00–2.94%) (Figure 1B,C).

### 2.2. B7 and TNFR Gene Families Are Over-Expressed in HCC

**Figure 2:**
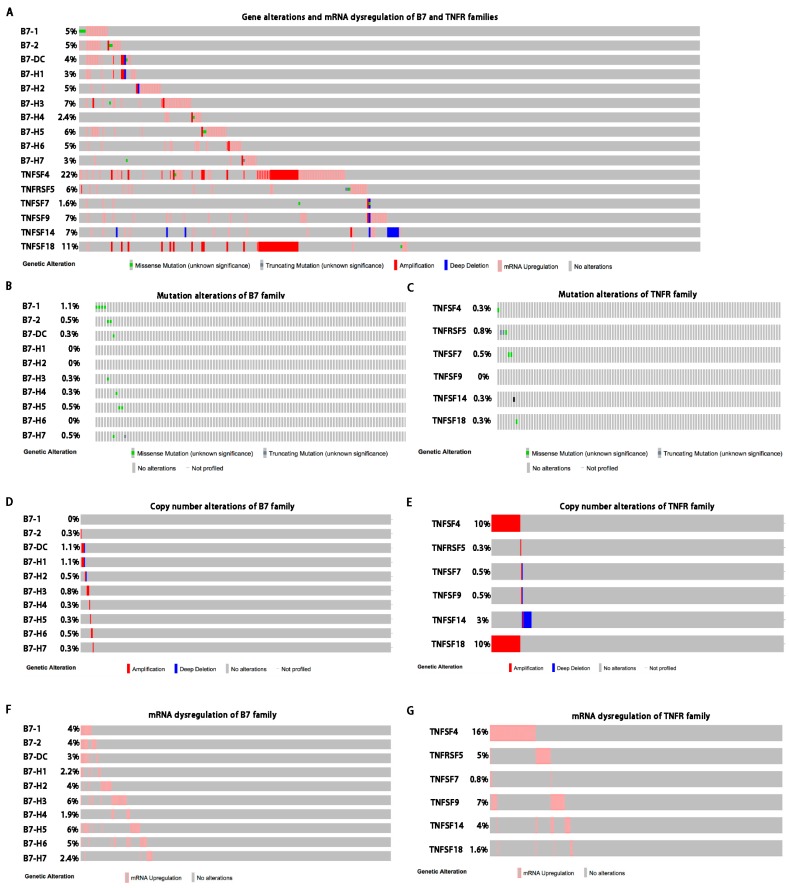
Gene alterations and mRNA dysregulations of B7 and TNFR family genes in HCC. (**A**) Gene alterations and mRNA dysregulations of B7 and TNFR family genes. (**B**) Mutation alterations of B7 family genes. (**C**) Mutation alterations of TNFR family genes. (**D**) Copy number alterations (CNA) of B7 family genes. (**E**) CNAs of TNFR family genes. (**F**) mRNA dysregulations of B7 family genes. (**G**) mRNA dysregulations of TNFR family genes.

Given the high frequencies of B7 and TNFR gene copy number variations (CNVs), we speculated that their expression might also be dysregulated in HCC. Thus, we next analyzed 371 sequenced HCC samples with data from The Cancer Genome Atlas (TCGA) by cBioPortal, and evaluated the mRNA alterations of the B7 and TNFR gene family members (Figure 2A). For each of the 10 B7 genes, mutations were neither observed nor present more than 1.1% of patients (Figure 2B). Similar frequencies of mutation were observed in the six TNFR genes (Figure 2C). The frequency of B7-DC and B7-H1 CNA was about 1.1%, while the frequency of TNFSF4 and TNFSF18 CNA was about 10.0%, (Figure 2D,E). Notably, B7 mRNA upregulation was observed in more than 23% (86/371) of HCC, while TNFR mRNA upregulation was upregulated in about 28% of cases (105/371). Obviously, all the genes were exclusively upregulated, ranging from 0.8% to 16.0%. The mRNA upregulation was relatively more common than mutations and CNAs in HCC (Figure 2F,G). Meanwhile, except for TNFSF4 and TNFSF18, the CNA rates of other TNFR genes were less than 3.0% (Figure 2E). The mRNA upregulation rates ranged from 0.8% to 16.0% in TNFR family (Figure 2F). Of note, the levels of TNFSF4 and TNFSF18 mRNA were increased in cases with CNAs including shallow deletions, diploid, copy number gains and amplification (Figures S1 and S2), which suggests that TNFSF4 and TNFSF18 mRNA levels could be partially regulated by copy number variation (CNV).

### 2.3. Correlation Analysis between Promoter DNA Methylation and mRNA Expression

**Figure 3:**
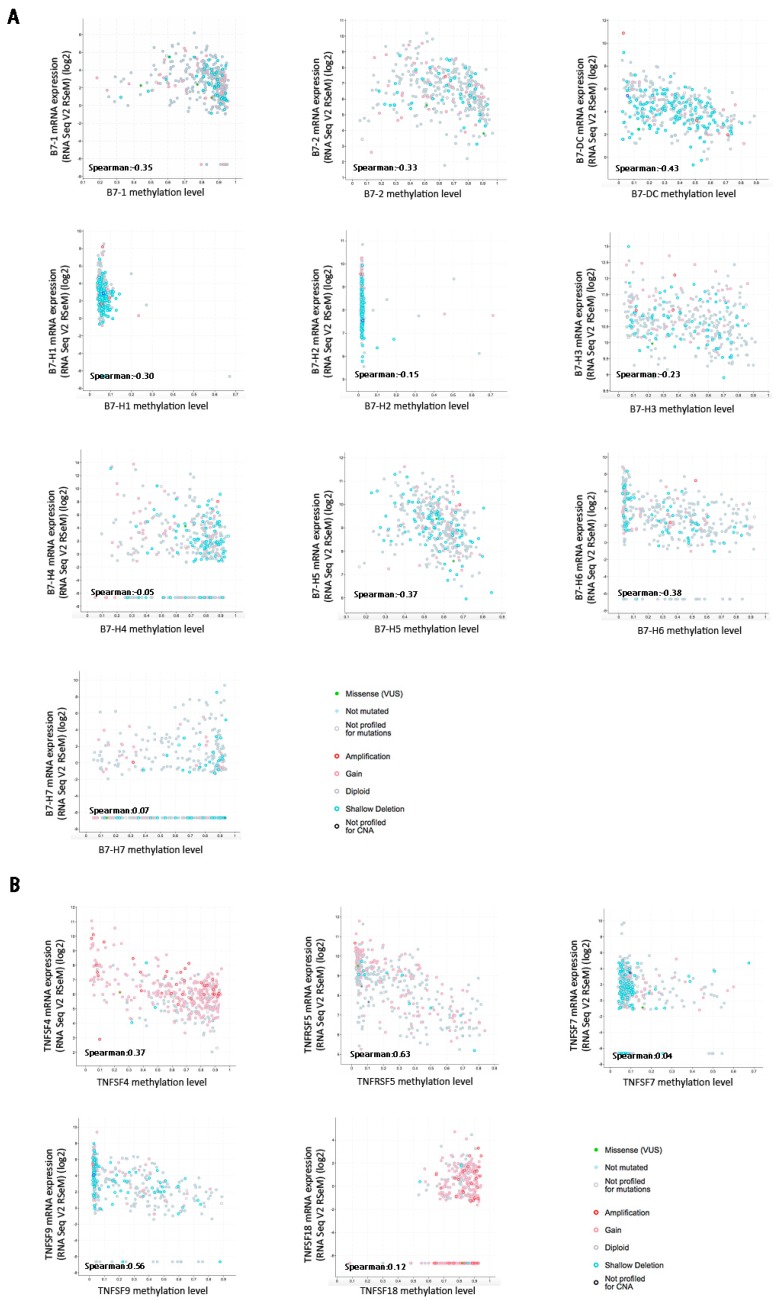
Spearman correlation between the promoter DNA methylation and mRNA expression of the B7 and TNFR families in HCC. (**A**) The mRNA levels of B7 family are negative correlated with their promoter methylated respectively in 371 HCC samples. (**B**) The mRNA levels of TNFR family are negative correlated with their promoter methylated respectively in 371 HCC samples.

Although B7 and TNFR proteins are constitutively expressed in HCC, the mechanisms of epigenetic modification responsible for mRNA dysregulation remain unclear. Therefore, we explored the promoter DNA methylation status of both family genes and investigated its correlation with the mRNA expression levels. By analyzing the DNA methylation levels and gene expression intensities (RNA-Seq V2 RSEM), we found that the mRNA levels of B7-1, B7-2, B7-DC, B7-H1, B7-H2, B7-H3, B7-H4, B7-H5, B7-H6, TNFSF4, TNFRSF5, TNFSF7, and TNFSF9 were negatively correlated with promoter methylation status (Figure 3). Notably, Spearman correlation analysis showed that B7-H6, B7-DC, TNFRSF5, and TNFSF9 mRNA levels were relatively strongly correlated with promoter DNA methylation (Spearman *r* = −0.38, −0.43, −0.63 and −0.56, respectively). These results suggest that epigenetical promoter DNA methylation may involve in regulating the ectopic expression of B7 and TNFR family members in HCC, especially B7-H6, B7-DC, TNFRSF5, and TNFSF9.

### 2.4. Prognostic Value of B7-H6 in HCC from the TCGA Cohort

**Figure 4:**
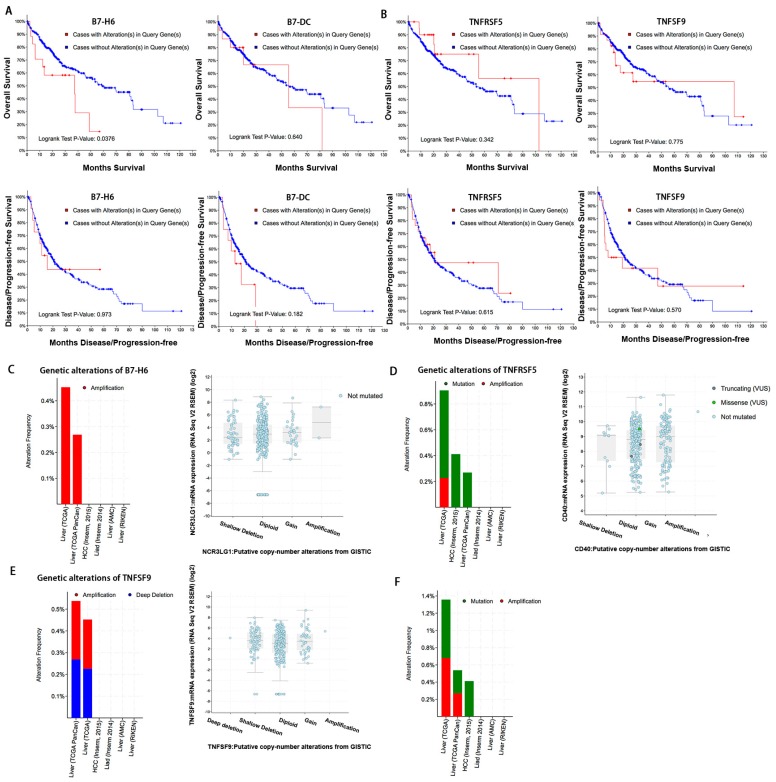
B7-H6 is a potential biomarker in HCC. (**A**) Overall survival (OS) and disease-free survival (DFS) of patients with B7-H6 and B7-DC alterations in HCC. (**B**) Overall survival (OS) and disease-free survival (DFS) of patients with TNFRSF5 and TNFSF9 alterations in HCC. (**C**) B7-H6 genetic alteration in six studies from cBioPortal and B7-H6 mRNA levels with copy number variations in HCC samples. (**D**) TNFRSF5 genetic alteration in six studies from cBioPortal and TNFRSF5 mRNA levels with copy number variations in HCC samples. (**E**) TNFSF9 genetic alteration in six studies from cBioPortal and TNFSF9 mRNA levels with copy number variations in HCC samples. (**F**) Both B7-H6 and TNFRSF5 gene alterations in six studies from cBioPortal. P values were determined using the log-rank test.

Given their ectopic expression in HCC, we next evaluated the clinical impact of the B7 and TNFR family members by accessing the overall survival (OS) and disease-free survival (DFS). We found that patients with B7-H6 alterations had significantly worse OS (*p* = 0.0376), but not B7-DC (Figure 4A). However, in addition to B7-H6, no obvious correlations with survival were observed in other B7 family members (Figures S3 and S4). We next foucused on B7-H6 and evaluated whether B7-H6 was genetically dysregulated in HCC data sets. We found that B7-H6 was amplified in HCC, although at relatively low frequencies (Figure 4C). We also found that B7-H6 mRNA levels which were strongly negatively correlated with promoter DNA methylation were increased gradually in cases with CNAs including shallow deletions, diploid, copy number gains, and amplification, suggesting that B7-H6 mRNA levels may also be regulated by copy number variation (Figure 4C).

In TNFR family, no obvious correlations with survival were observed including TNFRSF5 and TNFSF9 (Figure 4B). Our findings showed that TNFRSF5 was amplified and mutated in HCC datasets (Figure 4D). TNFSF9 was amplified and deep deleted in HCC datasets (Figure 4E). However, TNFRSF5 mRNA levels increased slightly in cases with gene copy number variations including shallow deletions, diploid, copy number gains, and amplification, but not TNFSF9 (Figure 4D,E). About 1.3% of patients had both B7-H6 and TNFRSF5 gene alterations (Figure 4F). As TNFRSF5 mRNA level was negatively correlated with promoter DNA methylation, it indicated that epigenetical DNA dysmethylation might play a critical role in regulating TNFRSF5 expression. These findings suggested that B7-H6 and TNFRSF5 could be promising targets in prediction of HCC survival.

### 2.5. B7-H6 Overexpression and Its Correlation with Clinical Parameters in HCC

**Figure 5:**
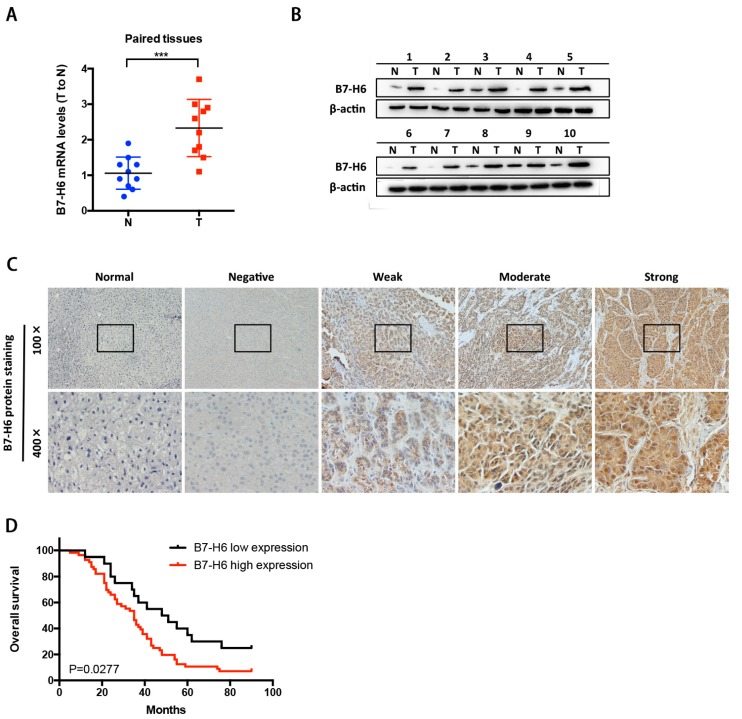
B7-H6 overexpression and its correlation with clinical parameters in HCC. (**A**) Detection of B7-H6 mRNA levels of in HCC (T) and adjacent non-tumor liver tissues (N) using real-time PCR. *** *p* < 0.001, Student’s *t*-test. (**B**) Detection of B7-H6 protein expression levels in HCC (T) and adjacent non-tumor liver tissues (N) using Western Blot. β-actin was used as control. (**C**) Cellular localization of B7-H6 using immunohistochemistry (IHC). Relatively negative, weak, moderate and strong staining images compared with normal tissues are shown. (**D**) The probability of overall survival (OS) was significantly different in B7-H6 low expression group and B7-H6 high expression group of the 74 patients (*p* < 0.05).

The overexpression of B7-H6 in tumor tissues was investigated by real-time PCR using 10 paired tissues (Figure 5A) and validated by western blot analysis (Figure 5B). To characterize the role of B7-H6 in HCC development and progression, the relationship between B7-H6 expression and clinicopathological parameters of HCC patients was analyzed by IHC in 74 specimens at the Fourth Military Medical University. We found that B7-H6 exhibited negative, weak, moderate and strong staining from HCC patients compared with non-tumor tissues (Figure 5C). B7-H6 expression levels were significantly associated with and T stages (*p* = 0.039) and M stages (*p* = 0.035) of HCC, but was not significantly associated with gender, age, tumor size, and pathological types (Table 1). Kaplan–Meier analysis showed that patients with high B7-H6 expression had a significantly lower overall survival rate (*p* = 0.0277) compared with those with low B7-H6 expression (Figure 5D).

### 2.6. Knockdown of B7-H6 Inhibits Adhesion, Proliferation, Migration, and Invasion of HCC Cells

**Figure 6:**
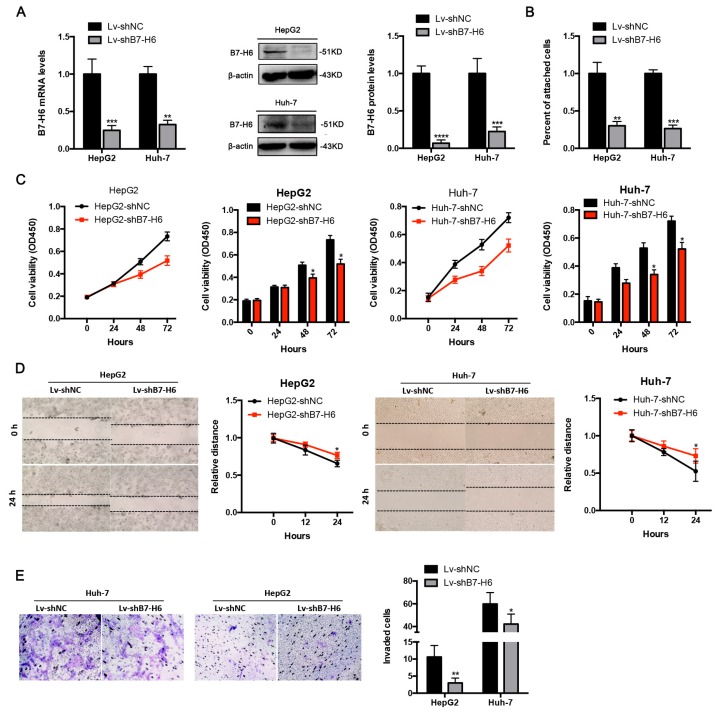
Knockdown of B7-H6 inhibits adhesion, proliferation, migration and invasion of HCC cells. (**A**) The knockdown rates of B7-H6 in HepG2 and Huh-7 by RT-qPCR and Western blot are shown. (**B**) Adhesion assays showed that adhesion capacity was reduced in HCC cells after transfected with shB7-H6 compared with negative control lentivirus. ** *p* < 0.01, *** *p* < 0.001, **** *p* < 0.0001, Student’s *t*-test. (**C**) The CCK-8 assays showed that proliferation was reduced in HCC cells after transfected with shB7-H6 compared with negative control lentivirus. * *p* < 0.05, Student’s *t*-test. (**D**) Wound healing assays were performed to examine the effect of B7-H6 knockdown on the migration of HCC cells (magnification 100×). * *p* < 0.05, Student’s *t*-test. (**E**) Transwell invasion assays showed that the number of crystal violet-stained cells was significantly decreased in the Lv-shB7-H6 group compared with that in the Lv-shNC group in both HepG2 and Huh-7 cells (at 24 h, magnification 100×, * *p* < 0.05 and * *p* < 0.05, respectively, Student’s *t*-test).

To explore the role of B7-H6 in malignant phenotypes and functions of HCC, we knocked down B7-H6 expression in both HepG2 and Huh-7 cells using shRNA via lentiviral infection. Knockdown efficiency was confirmed by real-time PCR and Western blot analysis (Figure 6A). B7-H6 knockdown significantly decreased adhesion rate of HepG2 and Huh-7 cells (Figure 6B). Proliferation rates of HepG2 and Huh-7 cells were significantly reduced compared with negative control cells (Figure 6C). Wound healing assays showed that B7-H6 knockdown significantly decrease the migration of HepG2 and Huh-7 cells (Figure 6D). In addition, invasion capacities of HepG2 and Huh-7 cells were significantly reduced compared with negative control cells (Figure 6E). Taken together, we demonstrated that B7-H6 promotes adhesion, proliferation, migration, and invasion of HCC cells.

### 2.7. Knockdown of B7-H6 in HCC Cells Inhibits Tumor Growth and Metastasis In Vivo

**Figure 7:**
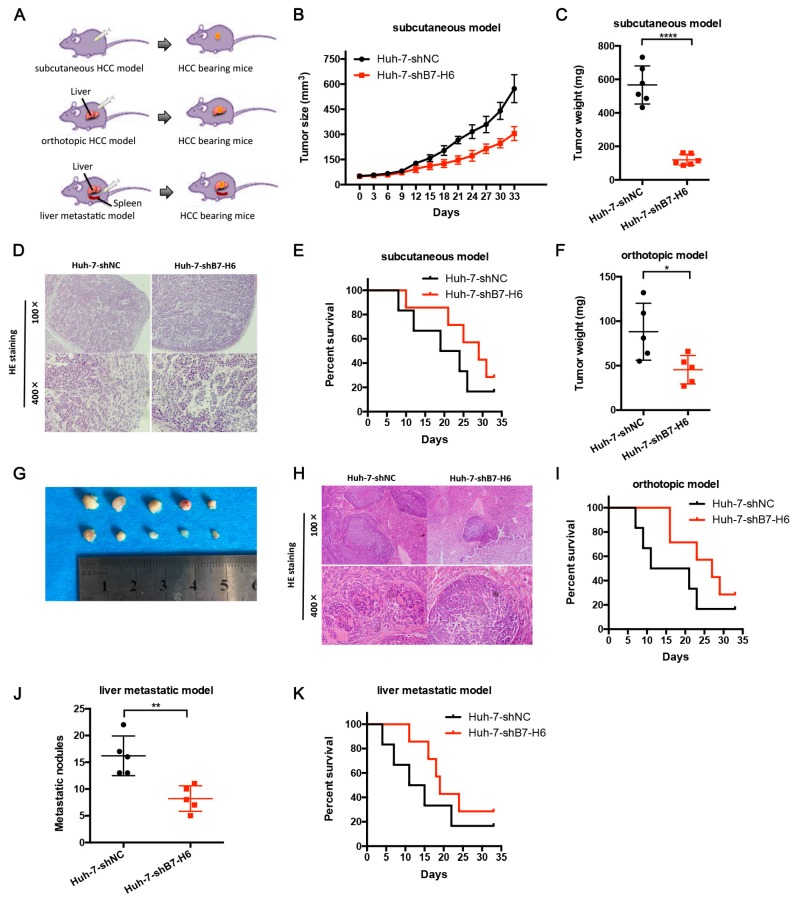
Knockdown of B7-H6 in HCC cells inhibits tumor growth and metastasis in vivo. (**A**) The subcutaneous model, orthotopic model, and liver metastatic model were shown respectively. (**B**,**C**) Huh7-shB7-H6 and Huh-7-shNC were subcutaneously injected into the left flank of the BALB/C nude mice. Tumor size (**B**) and weight (**C**) of two groups are shown. **** *p* < 0.0001, Student’s *t*-test. (**D**) Mice were sacrificed and the tumors were isolated. Representative H&E staining of tumor tissues is shown. (**E**) Survival rates of two subcutaneous groups are shown. (**F**) Huh7-shB7-H6 and Huh-7-shNC were orthotopically injected into the left lobe of the BALB/C nude mice. Tumor weight of two groups is shown. * *p* < 0.05, Student’s *t*-test. (**G**) Isolated tumors from liver lobes were shown. (**H**) Representative H&E staining of orthotopic tumor tissues is shown. (**I**) Survival rates of two orthotopic groups are shown. (**J**) Numbers of liver metastatic nodules are shown. ** *p* < 0.01, Student’s *t*-test. (**K**) Survival rates of two liver metastatic groups are shown.

To investigate the effect of B7-H6 on tumorigenicity and metastasis in vivo, we established three different xenograft HCC models in BALB/C nude mice, namely subcutaneous models, orthotopic models and liver metastatic models (Figure 7A). For the subcutaneous model, we found that the tumor growth was significantly inhibited in the Huh-7-shB7-H6 group compared with that of Huh-7-shNC group (Figure 7B,C). Histological examinations showed that there was no significant morphological difference between two groups in xenografted tumors (Figure 7D). The survival rate of Huh-7-shB7-H6 group was significantly higher than that of Huh-7-shNC group (Figure 7E). To better simulate tumorigenesis of HCC, we further constructed orthotopic models. Consistent results were observed in tumor growth and mouse survival (Figure 7F–I). At last, we constructed liver metastatic models to verify the role of B7-H6 in metastasis in vivo. Significantly decreased number of metastatic nodes in liver from spleen was observed in Huh-7-shB7-H6 group compared with those of Huh-7-shNC group (Figure 7J). Knockdown of B7-H6 significantly increased the survival rates of metastatic HCC-bearing mice (Figure 7K). Taken together, these results suggest that B7-H6 promotes HCC growth and metastasis in vivo.

### 2.8. B7-H6 Suppresses Tumor Progression and is Associated with Tumor Immunity

**Figure 8:**
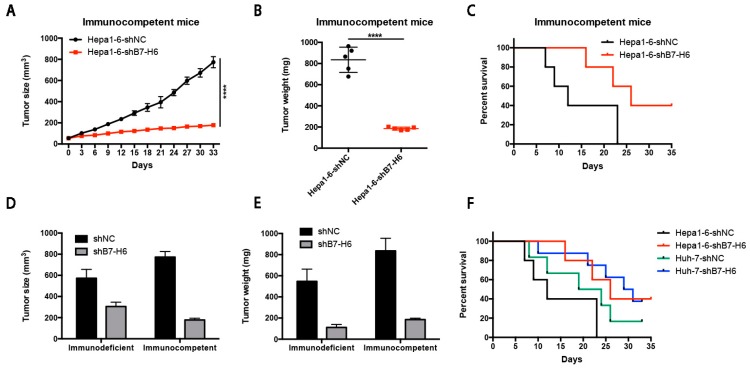
B7-H6 suppresses tumor progression and is associated with tumor immunity. (**A**,**B**) Hepa1-6-shB7-H6 and Hepa1-6-shNC were subcutaneously injected into the left flank of the C57BL/6 mice. Tumor size (**A**) and weight (**B**) of two groups are shown. **** *p* < 0.0001, Student’s *t*-test. (**C**) Survival rates of two subcutaneous groups are shown. (**D**,**E**) Tumor size and weight of subcutaneous tumors in immunocompetent and immunodeficient mice are shown. (**F**) Survival rates of four subcutaneous groups are shown.

Since we hypothesized that B7-H6 could be a new immunotherapy target, we next injected murine Hepa1-6-shB7-H6 cells subcutaneously into C57BL/6 mice (immunocompetent mice). B7-H6 depletion inhibited tumor growth and prolonged overall survival compared to those in control animals (Figure 8A–C). Moreover, significant difference in tumor growth and overall survival was observed in immunocompetent mice compared with immunodeficient mice (Figure 8D–F).

### 2.9. Knockdown of B7-H6 in HCC Cells Inhibits MMP-9 Expression and STAT3 Activation

**Figure 9:**
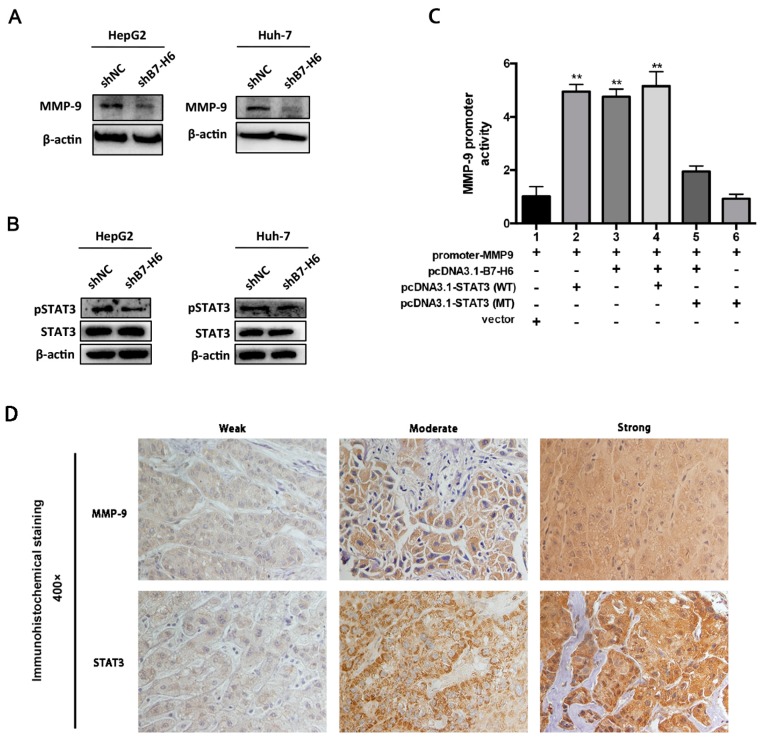
Knockdown of B7-H6 in HCC cells inhibits MMP-9 expression and STAT3 activation. (**A**) Expression of MMP-9 was reduced in HepG2-shB7-H6 and Huh-7-shB7-H6 cells compared with negative control cells, respectively. (**B**) Knockdown of B7-H6 inhibited phosph-STAT3 (p-STAT3) expression in both Huh-7 and HepG2 cells. (**C**) The activity of the MMP-9 promoter was tested by Luciferase reporter assay in Huh-7 cells. MMP-9/Luc promoter-reporter construct was transfected into Huh-7 cells alone or in combination with B7-H6-pcDNA3.1, or STAT3 (WT)-pcDNA3.1, or STAT3 (MT)-pcDNA3.1 expression plasmids, or pcDNA3.1 empty vector. After transfection for 48 h, the luciferase activity was measured. ** *p* < 0.01, Student’s *t*-test. (**D**) Representative immunohistochemical staining of MMP-9 and STAT3 in HCC tumor specimens.

We also investigated the expression of MMP-9, a crucial regulator in cell migration and invasion, and found that expression of MMP-9 was reduced in HepG2-shB7-H6 and Huh-7-shB7-H6 cells compared with negative control cells, respectively (Figure 9A). As STAT3 is a well-known transcriptional factor in regulating tumor metastasis, we further investigate whether B7-H6 promotes tumor invasion and metastasis via STAT3 activation. We found that knockdown of B7-H6 inhibited phosph-STAT3 (p-STAT3) expression in both Huh-7 and HepG2 cells (Figure 9B). To further detect whether B7-H6-promoted MMP-9 induction was mediated through STAT3, an MMP-9/Luc promoter-reporter construct was transfected into Huh-7 cells alone or in combination with B7-H6 or STAT3 expression plasmids. We observed that either B7-H6 or STAT3 alone induced luciferase activity in cells. However, no further enhancement was observed in the co-transfection of B7-H6 and STAT3 expression plasmids (Figure 9C and Figure S5). In addition, we further performed immunohistochemical staining of MMP-9 and STAT3 in HCC tumor specimens (Figure 9D). These data converged to show that B7-H6 and STAT3 play functional overlapping roles on enhancing the MMP-9 promoter activity in HCC.

## 3. Discussion

In cancer, dysregulation of co-inhibitory B7 and TNFR family molecules is associated with attenuated anti-tumor immunity and enhanced immune evasion. In this study, we portrayed the comprehensive perspectives and clinical relevance of immune co-stimulator B7 and TNSF family genes in HCC. Given the promising results from clinical trials assessing the immunotherapy of advanced or treatment-refractory cancer with immune checkpoint inhibitors such as anti-PD-1/PD-L1, the B7 and TNFR family members may be closely evaluated as potential immunotherapeutic targets in HCC. Here, we provide a systematic overview of 10 B7 family members and 6 TNFR family members respectively, under a unified nomenclature according to previous studies. Both mutations and CNAs were observed in B7 and TNFR family genes. Intriguingly, we found that their mRNA dysregulation was more common and associated with promoter DNA methylation. Furthermore, the alterations of B7-H6 were correlated with unfavorable overall survival, which may serve as a promising candidate for HCC immunotherapy. We found that B7-H6 overexpression was associated with aggressive clinicopathologic features and poor prognosis in HCC. Downregulation of B7-H6 in HCC cells significantly inhibited cell adhesion, proliferation, migration, and invasion. Knockdown of B7-H6 in HCC cells inhibited tumor growth and metastasis in vivo. Furthermore, B7-H6 promtes MMP-9 expression and STAT3 activation in HCC cells. B7-H6 and STAT3 perform functional overlapping roles on enhancing the MMP-9 promoter activity in HCC. Hence, B7-H6 might be a potential target for HCC treatment.

Despite that checkpoint blockade has provided prolonged clinical benefits, but this benefit remains limited to only a small subset of patients [14,15,16]. Therefore, it has been much warranted to explore novel and effective candidates for cancer immunotherapy. In this study, we performed a comprehensive genetic analysis of B7 and TNFR family genes and found that both of the family genes exhibited mutation, amplification or deletion in HCC, which is consistent with previous studies in breast cancer, colorectal cancer, and head and neck cancer [17,18,19]. Overall, both B7 and TNFR gene alterations were observed in about 16% of patients and might contribute to activating or inhibiting immune response in HCC, although the mutation and CNA rates were relatively low. Besides, we found that the B7-H6 alterations were associated with poor overall survival, demonstrating its potential role as a predictive biomarker of immunotherapy regimens. Taken together, our findings suggested that targeting B7-H6 might represent an attractive and promsing therapeutic approach against HCC.

The B7 family and their cognate receptors are the major drivers of T cell co-stimulated activation and co-inhibited inactivation. In addition to the B7 family, the TNFR superfamily members possess the indispensible properties for immune regulation in cancer. Based on the fundamental mechanisms of the TNFR family members, the translational investigation has brought about the research and development of new therapeutic immune agents besides CTLA-4 and PD-L1/PD-1 antibodies, such as dacetuzumab (humanized TNFRSF5 monoclonal antibody) and urelumab (fully human TNFRSF9 monoclonal antibody) [8]. Various clinical trials have demonstrated that blockade of TNFRSF9 altered the tumor microenvironment and minimized systemic exposure [20]. However, we found that alterations in the six TNFR family members were relatively less common and were not significantly correlated with prognosis. We speculated that the role of the TNFR family in anti-tumor immunity might not be as important as that of the B7 family in HCC. Further basic studies are warranted to explore the therapeutic value of the TNFR family members.

The limitation of this study should be acknowledged. First, although the the data from cBioPortal for Cancer Genomics enrolled large-scale cancer genomic projects, more specialized and representative cohorts should be conducted to illustrate the landscape of the B7 and TNFR families in HCC. Second, with the exception of B7-H6, the protein expression of other B7 and TNFR family genes was not validated by immunohistochemistry in HCC specimens. Moreover, more large-scale prospective studies and multicenter clinical trials are much warranted to further validate our findings. Nevertheless, this study steps forward to establishing the comprehensive genetic and immune landscape of the B7 and TNFR families in HCC, and these findings could help provide potential candidates to stratify patients based on response to immune checkpoint therapy.

In conclusion, our study provided a systematic analysis of B7 and TNFR family gene alterations in HCC. Notably, B7-H6 alteration is significantly associated with poor overall survival, and its mRNA dysregulation plausibly correlates to both gene amplification and DNA methylation. B7-H6 overexpression was associated with aggressive clinicopathologic features and poor prognosis in HCC. Downregulation of B7-H6 in HCC cells significantly inhibited cell adhesion, proliferation, migration, and invasion. Knockdown of B7-H6 in HCC cells inhibited tumor growth and metastasis in vivo. To our knowledge, this is the first study on the immune molecular landscape of the pivotal B7 and TNFR families in HCC, thereby facilitating the development of novel promising immunotherapy against HCC.

## 4. Materials and Methods

### 4.1. Cell Culture and Gene Silencing

HepG2, Huh-7, Hepa1-6, and HEK-293 cells were purchased from Institute of Cell Biology (Shanghai, China). Cells were cultured in RPMI 1640 medium (11875-093, Gibco, Waltham, MA, USA), containing 10% fetal bovine serum (FBS), 1% penicillin/streptomycin and 2% L-glutamine at 37 °C in a humidified atmosphere of 5% CO2. Lentivirus was used to establish stable B7-H6 knockdown HCC cells, namely shNC and shB7-H6 cells, respectively. The shNC cells were used as control mock.

### 4.2. Patients and Specimens

A total of 74 cases of human HCC tissuses from patients who underwent surgical dissections were enrolled in this study, and were all diagnosed with HCC. The surgical samples were supplied by the Xijing Hospital at the Fourth Military Medical University. Histopathological sections of each patient by HE staining were reviewed by two independent pathologists to evaluate the grades and TNM stages. The TNM stages were assessed based on the American Joint Committee on Cancer (AJCC) TNM classification for HCC. Tumor differentiation grading was classified by the Edmondson grading system. Eligible patients in this study were those with histopathological confirmation, sufficient samples for IHC staining and qRT-PCR, and complete clinical and outcome statistics. Through screening electronic database by follow-up, we retrospectively collected those patients with adequate follow-up information. Overall survival (OS) rates were calculated from the date of surgery to death or until the last follow-up (censored).

### 4.3. Integrative Alteration Analysis of B7 and TNFR Family Members

Integrative alteration analysis of B7 and TNFR family members on HCC was conducted using the cBioPortal for Cancer Genomics database and TCGA with more than 1000 patients’ details [21]. Six large-scale studies were enrolled to assess the frequency of B7 and TNFR gene alterations including mutations, deletions, and amplifications. Mutations included missense mutations and truncating mutations. Whole-exome sequencing was utilized to identify gene mutations. Mutations were validated by targeted re-sequencing and interrogation of RNA for expression of the mutated alleles, which included truncating, inframe, and missense mutations. Missense mutations are point mutations changing a single nucleotide that results in the substitution of a different amino acid and a nonfunctional protein. Truncating mutations are point mutations in the genetic code that generate one stop codon and interrupt protein translation. Any insertion or deletion that is evenly divisible by three is termed an inframe mutation, which would not disrupt the reading frame, given the triplet nature of gene expression by codons. Furthermore, we evaluated the genomic alterations including mRNA dysregulation and promoter methylation of both families across 371 sequenced HCC samples with complete TCGA data, as queried with cBioPortal. All mRNA data were assayed by mRNA-seq and gene expression values were represented as RNA-Seq by expectation maximization (RSEM) data normalized within each sample to the upper quartile of total reads [22].

### 4.4. Association of B7 and TNFR Families with Prognosis in HCC Patients

The clinicopathological information and survival data were obtained from the cBioPortal for Cancer Genomics database and TCGA. B7 and TNFR family members’ mRNA dysregulations were assessed to correlate with the prognosis of patients with HCC. We performed receiver operating characteristic (ROC) curve analysis to determine cut-off value of mRNA.

### 4.5. Immunohistochemistry (IHC)

4µm thick sections were deparaffinized, followed by antigen retrieval with 10 mmol/L citrate buffer (ZLI-9605, Zhongshan Jinqiao Co., Beijing, China) at pH6.0. Endogenous peroxidase activity was blocked with methanol containing 3% hydrogen peroxide for 15 min. After serum block, sections were incubated with anti-B7-H6 (ab121794, Abcam, Cambridge, MA, USA), anti-MMP9 (ab38898, Abcam, Cambridge, MA, USA), anti-STAT3 (ab119352, Abcam, Cambridge, MA, USA) at 4 °C overnight. Following incubation, immunoperoxidase staining was carried out by a streptavidin-peroxidase-3,3′-diaminobenzidine kit (SPN-9001, Zhongshan Jinqiao Co., Beijing, China) to visualize the target proteins. Hematoxylin was utilized to counterstain the nuclei. Isotype control and negative control were used to evaluate specificity of all antibodies. Two independent pathologists who were blinded to the experiment evaluated the sections. Major evaluation parameters include the intensity and density of positive cells.

### 4.6. Real-Time Quantitative PCR and Data Analysis

RT-PCR was conducted to confirm the knockdown of B7-H6 expression at the mRNA level in HepG2 and Huh-7 cells. Total RNA was extracted by utilization of Total RNA Kit II (R6934-01, Omega Bio-tek, Norcross, GA, USA) following manufacturer’s instructions, and then reversely transcribed to cDNA by use of the PrimeScript RT Reagent Kit (TaKaRa). The SYBR Premix Ex Taq II Kit (RR037A, TaKaRa) was used in qRT-PCR. Human GAPDH mRNA was used as an internal control. PCR Primers were synthesized as follows: GAPDH forward primer, 5′- CTGGGCTACACTGAGCACC-3′, and reverse primer, 5′-AAGTGGTCGTTGAGGGCAATG-3′; B7-H6 forward primer, 5′-CTCCTGATTCTGCT GTGGGC-3′, and reverse primer, 5′-GTCGGAATGCCTCTTGGTGA -3′. Real-time quantitative PCR was performed with a Stratagene M × 3005P Multiplex Quantitative PCR System (Agilent Technologies, Santa Clara, CA, USA).

### 4.7. Western Blot Analysis

Cells were harvested and then treated with RIPA lysis buffer containing phenylmethylsulfonyl floride (KGP610, KeyGen BioTech, Nanjing, China), protease inhibitors (KGP603, KeyGen BioTech, Nanjing, China) and phosphatase inhibitors (KGP602, KeyGen BioTech, Nanjing, China). Lysates were incubated on ice for 30 min, centrifuged (14,000 rpm, 20 min, 4 °C) and were quantified by the BCA Protein Assay Kit (23225, Thermo Fisher Scientific, Waltham, MA, USA). The supernatants were subjected to the subsequent SDS-PAGE. Protein samples were boiled for 5 min and then loaded to 12% SDS-PAGE gel by equal amounts. Gels were transferred to PVDF membranes (IPVH00010, Millipore, Temecula, CA, USA). After blockade in 5% non-fat milk in TBST (50 mM Tris/HCl, pH 7.4, 150 mM NaCl, 0.1% Tween-20) for 1 h, the membrane was incubated with the designated primary antibodies (anti-B7-H6 ab121794, anti-MMP9 ab38898, anti-STAT3 ab119352, anti-pSTAT3 ab76315, Abcam; anti-β-actin, M1210-2, HuaBio, Hangzhou, China) at 4 °C overnight. The images were developed after incubation with the secondary antibodies at room temperature for 1 h. Bands were visualized by Chemidoc Touch (Bio-Rad, Hercules, CA, USA).

### 4.8. Cell Adhesion Assay

96-well plates were coated 100 μL Matrigel (354234, BD Biosciences, San Jose, CA, US) per well at 4 °C overnight. The plates were washed twice with PBS and blocked with 2% BSA for 30 min at 37 °C. HCC cells transfected with shB7-H6 or shNC lentivirus were collected and added into each gel-coated well and incubated for 1 h at 37 °C in 5% CO_2_. Unattached cells were gently washed away with PBS. The attached cells were fixed with 4% formaldehyde, stained with 0.2% crystal violet for 20 min, and destained with distilled water. After exposure in air for 24 h, solubilized in lysis buffer for 30 min and quantified by the microplate reader (BioTek, Winooski, VT, USA).

### 4.9. Cell Proliferation Assay

HCC cells transfected with shB7-H6 or shNC lentivirus were seeded into 96-well plates at a density of 4 × 10^4^ cells per well. Cell Counting Kit-8 (CCK-8, C0038, Beyotime, Beijing, China) was used to determine the OD value at a wavelength of 450 nm after 1 h according to the manufacturer’s instructions. Cell proliferation was examined every 24 h over a period of three days. All experiments were performed in triplicate, and the mean proliferation rate was adopted.

### 4.10. Wound Healing Assay

HCC cells transfected with shB7-H6 or shNC lentivirus were cultured in 6-well plates. A straight wound area was created using a 100-μL pipette tip when cells reached a confluence of 90–100%. Then the scraped cells were washed with PBS twice and cultured in fresh serum-free RPMI 1640 medium (Gibco, Waltham, MA, USA), at 37 °C in a humidified atmosphere of 5% CO2. Images were acquired at 0, 12 and 24 h for at least five non-overlapping fields. All experiments were performed in triplicate, and the mean proliferation rate was adopted.

### 4.11. Transwell Invasion Assay

The upper chamber of Transwell inserts (8 μm) was coated with 100 μL of Matrigel (diluted 1:4 in serum-free RPMI 1640 medium) and was incubated at 37 °C for 2 h. For invasion assay, 3 × 10^5^ cells transfected with shB7-H6 or shNC in 200 μL serum-free RPMI 1640 medium were added to the upper chamber of the coated inserts (Merck Millipore, Temecula, CA, USA) of the 24-well plates with 500 μL of RPMI 1640 medium containing 10% FBS in the bottom chamber. After incubation at 37 °C for 24 h, the non-invading cells were removed slightly from the upper chamber with a cotton-tipped swab. The inserts were rinsed with PBS and the migrated cells were fixed with 95% ethanol with 0.2% crystal violet for 30 min. The invaded cells were counted in five randomly selected fields. All experiments were performed in triplicate, and the mean proliferation rate was adopted.

### 4.12. Subcutaneous HCC Model in Nude Mice

For subcutaneous HCC model, 5 × 10^6^ Huh-7-shB7-H6 or Huh-7-shNC cells were injected into the left flanks of BALB/C nude mice. Tumors were measured with a caliper and calculated with the formula length × width × depth × 0.52 (cubic millimeters). The volume of the tumor was measured every third day for 33 days. The survival time was also calculated for each mouse. Then mice were sacrificed, and their tumors were harvested and weighed. Livers and tumors were dissected, fixed with 10% formalin and washed with 75% ethanol. Then the tissues were prepared for standard histological examination. Animal studies were approved by the Laboratory Animal Research Center of the Fourth Military Medical University.

### 4.13. Orthotopic HCC Model in Nude Mice

For orthotopic implantation, 5 × 10^6^ Huh-7-shB7-H6 or Huh-7-shNC cells were injected into the left lobe of the livers of BALB/c nude mice via a microsyringe through an 8-mm transverse incision under anesthesia. The survival time was calculated for 33 consecutive days. Then mice were sacrificed, and their livers and tumors were dissected, fixed with 10% formalin and washed with 75% ethanol. Then the tissues were prepared for standard histological examination.

### 4.14. Metastatic HCC Model in Nude Mice

For establishement of metastatic HCC model, 5 × 10^6^ Huh-7-shB7-H6 or Huh-7-shNC cells were injected into the spleens of BALB/c nude mice via a microsyringe through an 8-mm transverse incision under anesthesia. The survival time was calculated for 33 consecutive days. Then mice were sacrificed, and their livers were dissected, fixed with 10% formalin and washed with 75% ethanol. Then the tissues were prepared for standard histological examination.

### 4.15. Subcutaneous HCC Model in Immune Competent Mice

For subcutaneous HCC model in immune competent mice, 2 × 10^6^ Hepa1-6-shB7-H6 or Hepa1-6-shNC cells were injected into the left flanks of C57BL/6 mice. Tumors were measured with a caliper and calculated with the formula length × width × depth × 0.52 (cubic millimeters). The volume of the tumor was measured every third day for 33 days. The survival time was also calculated for each mouse. Then mice were sacrificed, and their tumors were harvested and weighed. Livers and tumors were dissected, fixed with 10% formalin, and washed with 75% ethanol. Then the tissues were prepared for standard histological examination.

### 4.17. Dual-Luciferase Reporter Assay

Huh-7 and 293T cells were transfected with firefly luciferase reporter gene constructs and hRluc-CMV vector (Promega, Madison, WI, USA). The MMP-9 promoter region was cloned by gene synthesis in according with coding sequence and was inserted into pGL4.10 luciferase vector (Promega). The MMP-9 promoter-reporter construct was then transfected into Huh-7 and 293T cells alone or in combination with the following vectors by using Lipofectamine 2000 (11668019, Invitrogen) as recommended by the manufacturer: B7-H6-pcDNA3.1, STAT3-pcDNA3.1 (Wide Type, WT), STAT3-pcDNA3.1 (Mutation Type, MT), and Vector-pcDNA3.1. In each transfection system, an empty vector was added to the reaction mixture to normalize the amount of total DNA. At 48 h post-transfection, Firefly and Renilla luciferase intensities were measured using the Dual-Luciferase Reporter Assay System (E1980, Promega) according to the manufacturer.

### 4.18. Statistics

The statistical analyses were performed by SPSS 23.0 (SPSS, Chicago, IL, USA) and GraphPad Prism 7 (GraphPad Software, La Jolla, CA, USA). Student’s *t*-test (two-tailed) was used to compare the means between two groups. The Pearson chi-square test was used to compare categorical variables, and Fisher’s exact test was used if indicated. The Spearman correlation test was conducted to assess the relationship between promoter methylation and mRNA expression. Overall survival was calculated using Kaplan–Meier analysis and compared by the log-rank test. The mRNA expression data were presented as mean ± SD. p values less than 0.05 were considered statistically significant (two-tailed).

## Figures and Tables

**Table 1:** Correlation between B7-H6 expression and clinical parameters in HCC.

Parameters	Cases	B7-H6 Expression Level		*p*
		<Median	>Median	
Gender				0.304
Male	49	12	37	
Female	25	9	16	
Age (years)				0.582
<55	39	10	29	
≥55	35	11	24	
Tumor size (cm)				0.388
≤5	51	16	35	
>5	23	5	18	
T stage				0.039
I+II	39	15	24	
III+IV	35	6	29	
M stage				0.013
M0	48	18	30	
M1	26	3	23	
Pathological type				0.368
Massive	47	15	32	
Nodular	27	6	21	

## Supplementary Materials

Supplementary materials can be found at http://www.mdpi.com/1422-0067/20/1/156/s1.

## References

[1] Prieto J., Melero I., Sangro B. (2015). Immunological landscape and immunotherapy of hepatocellular carcinoma. Nat. Rev. Gastroenterol. Hepatol..

[2] Sharma P., Hu-Lieskovan S., Wargo J.A., Ribas A. (2017). Primary, Adaptive, and Acquired Resistance to Cancer Immunotherapy. Cell.

[3] Pitt J.M., Vetizou M., Daillere R., Roberti M.P., Yamazaki T., Routy B., Lepage P., Boneca I.G., Chamaillard M., Kroemer G. (2016). Resistance Mechanisms to Immune-Checkpoint Blockade in Cancer: Tumor-Intrinsic and -Extrinsic Factors. Immunity.

[4] Gao B., Jeong W.I., Tian Z. (2008). Liver: An organ with predominant innate immunity. Hepatology.

[5] Sharma P., Allison J.P. (2015). The future of immune checkpoint therapy. Science.

[6] Zhu J., de Tenbossche C.G.P., Cane S., Colau D., van Baren N., Lurquin C., Schmitt-Verhulst A.M., Liljestrom P., Uyttenhove C., Van den Eynde B.J. (2017). Resistance to cancer immunotherapy mediated by apoptosis of tumor-infiltrating lymphocytes. Nat. Commun..

[7] Chen L., Flies D.B. (2013). Molecular mechanisms of T cell co-stimulation and co-inhibition. Nat. Rev. Immunol..

[8] Assal A., Kaner J., Pendurti G., Zang X. (2015). Emerging targets in cancer immunotherapy: Beyond CTLA-4 and PD-1. Immunotherapy.

[9] Schildberg F.A., Klein S.R., Freeman G.J., Sharpe A.H. (2016). Coinhibitory Pathways in the B7-CD28 Ligand-Receptor Family. Immunity.

[10] Ward-Kavanagh L.K., Lin W.W., Sedy J.R., Ware C.F. (2016). The TNF Receptor Superfamily in Co-stimulating and Co-inhibitory Responses. Immunity.

[11] Pardoll D.M. (2012). The blockade of immune checkpoints in cancer immunotherapy. Nat. Rev. Cancer.

[12] Eroglu Z., Zaretsky J.M., Hu-Lieskovan S., Kim D.W., Algazi A., Johnson D.B., Liniker E., Ben K., Munhoz R., Rapisuwon S. (2018). High response rate to PD-1 blockade in desmoplastic melanomas. Nature.

[13] Gordon S.R., Maute R.L., Dulken B.W., Hutter G., George B.M., McCracken M.N., Gupta R., Tsai J.M., Sinha R., Corey D. (2017). PD-1 expression by tumour-associated macrophages inhibits phagocytosis and tumour immunity. Nature.

[14] Topalian S.L., Hodi F.S., Brahmer J.R., Gettinger S.N., Smith D.C., McDermott D.F., Powderly J.D., Carvajal R.D., Sosman J.A., Atkins M.B. (2012). Safety, activity, and immune correlates of anti-PD-1 antibody in cancer. N. Engl. J. Med..

[15] Topalian S.L., Drake C.G., Pardoll D.M. (2012). Targeting the PD-1/B7-H1(PD-L1) pathway to activate anti-tumor immunity. Curr. Opin. Immunol..

[16] Le D.T., Durham J.N., Smith K.N., Wang H., Bartlett B.R., Aulakh L.K., Lu S., Kemberling H., Wilt C., Luber B.S. (2017). Mismatch repair deficiency predicts response of solid tumors to PD-1 blockade. Science.

[17] Xu Z., Shen J., Wang M.H., Yi T., Yu Y., Zhu Y., Chen B., Chen J., Li L., Li M. (2016). Comprehensive molecular profiling of the B7 family of immune-regulatory ligands in breast cancer. Oncoimmunology.

[18] Chen Y.P., Zhang J., Wang Y.Q., Liu N., He Q.M., Yang X.J., Sun Y., Ma J. (2017). The immune molecular landscape of the B7 and TNFR immunoregulatory ligand-receptor families in head and neck cancer: A comprehensive overview and the immunotherapeutic implications. Oncoimmunology.

[19] Tang J., Jiang W., Liu D., Luo J., Wu X., Pan Z., Ding P., Li Y. (2018). The comprehensive molecular landscape of the immunologic co-stimulator B7 and TNFR ligand receptor families in colorectal cancer: Immunotherapeutic implications with microsatellite instability. Oncoimmunology.

[20] Chester C., Sanmamed M.F., Wang J., Melero I. (2018). Immunotherapy targeting 4-1BB: Mechanistic rationale, clinical results, and future strategies. Blood.

[21] Cerami E., Gao J., Dogrusoz U., Gross B.E., Sumer S.O., Aksoy B.A., Jacobsen A., Byrne C.J., Heuer M.L., Larsson E. (2012). The cBio cancer genomics portal: An open platform for exploring multidimensional cancer genomics data. Cancer Discov..

[22] Palmer C., Diehn M., Alizadeh A.A., Brown P.O. (2006). Cell-type specific gene expression profiles of leukocytes in human peripheral blood. BMC Genomics.

