# Supplementary material for: Alterations of the Immunologic Co-Stimulator B7 and TNFR Families Correlate with Hepatocellular Carcinoma Prognosis and Metastasis by Inactivating STAT3
Source: Int J Mol Sci. 2019 Jan 3;20(1):156. doi: 10.3390/ijms20010156 (PMC6337299; doi:10.3390/ijms20010156)
Supplement: Supplementary file 1 [file ijms-20-00156-s001.zip › supplementary figure 2.doc]

**Figure S2. Plot analysis of the mRNA and CNA alteration.** Increased TNFSF18 mRNA levels with copy number variations in HCC patients.
