# Supplementary material for: Alterations of the Immunologic Co-Stimulator B7 and TNFR Families Correlate with Hepatocellular Carcinoma Prognosis and Metastasis by Inactivating STAT3
Source: Int J Mol Sci. 2019 Jan 3;20(1):156. doi: 10.3390/ijms20010156 (PMC6337299; doi:10.3390/ijms20010156)
Supplement: Supplementary file 1 [file ijms-20-00156-s001.zip › supplementary figure 3.doc]

**Figure S3. Overall survival (OS) of patients with B7 and TNFR gene alterations in HCC.** (A) Months survival and B7-1. (B) Months survival and B7-2. (C) Months survival and B7-H1. (D) Months survival and B7-H2. (E) Months survival and B7-H3. (F) Months survival and B7-H4. (G) Months survival and B7-H5. (H) Months survival and B7-H7. (I) Months survival and TNFSF4. (J) Months survival and TNFSF7. (K) Months survival and TNFRSF14. (L) Months survival and TNFSF18.
