# Supplementary material for: Alterations of the Immunologic Co-Stimulator B7 and TNFR Families Correlate with Hepatocellular Carcinoma Prognosis and Metastasis by Inactivating STAT3
Source: Int J Mol Sci. 2019 Jan 3;20(1):156. doi: 10.3390/ijms20010156 (PMC6337299; doi:10.3390/ijms20010156)
Supplement: Supplementary file 1 [file ijms-20-00156-s001.zip › supplementary figure 4.doc]

**Figure S4. Disease-free survival (DFS) of patients with B7 and TNFR gene alterations in HCC.** (A) Months disease/progression-free and B7-1. (B) Months disease/progression-free and B7-2. (C) Months disease/progression-free and B7-H1. (D) Months disease/progression-free and B7-H2. (E) Months disease/progression-free and B7-H3. (F) Months disease/progression-free and B7-H4. (G) Months disease/progression-free and B7-H5. (H) Months disease/progression-free and B7-H7. (I) Months disease/progression-free and TNFSF4. (J) Months disease/progression-free and TNFSF7. (K) Months disease/progression-free and TNFRSF14. (L) Months disease/ progression-free and TNFSF18.
