# Supplementary material for: Alterations of the Immunologic Co-Stimulator B7 and TNFR Families Correlate with Hepatocellular Carcinoma Prognosis and Metastasis by Inactivating STAT3
Source: Int J Mol Sci. 2019 Jan 3;20(1):156. doi: 10.3390/ijms20010156 (PMC6337299; doi:10.3390/ijms20010156)
Supplement: Supplementary file 1 [file ijms-20-00156-s001.zip › supplementary figure 5.doc]

**Figure S5. B7-H6 and STAT3 play functional overlapping roles on enhancing the MMP-9 promoter activity in HEK-293 cells.** An MMP-9/Luc promoter-reporter construct was transfected into HEK-293 cells alone or in combination with B7-H6 or STAT3 expression plasmids. Either B7-H6 or STAT3 alone induced luciferase activity in cells. No further enhancement was observed in the co-transfection of B7-H6 and STAT3 expression plasmids.
